# Opioid and benzodiazepine dispensing and co-dispensing patterns among commercially insured pregnant women in the United States, 2007–2015

**DOI:** 10.1186/s12884-021-03787-5

**Published:** 2021-05-03

**Authors:** Danya M. Qato, Aakash Bipin Gandhi

**Affiliations:** 1 Department of Pharmaceutical Health Services Research, University of Maryland School of Pharmacy, Baltimore, MD USA; 2 Department of Epidemiology and Public Health, University of Maryland School of Medicine, Baltimore, MD USA

**Keywords:** Pregnant women, Commercially insured, Opioid, Benzodiazepine, Dispensing, Trend analysis

## Abstract

**Background:**

Little is known about benzodiazepine and opioid-benzodiazepine co-dispensing patterns among pregnant women. Understanding these patterns is necessary to mitigate high-risk medication use during pregnancy. Our objective in this analysis was to evaluate opioid and benzodiazepine dispensing and co-dispensing patterns among commercially insured pregnant women in the United States.

**Methods:**

This retrospective study used a 10% random sample of commercially insured enrollees from the IQVIA™ Adjudicated Health Plan Claims Data from 2007 to 2015. The study included women (12–55 years of age) with completed pregnancies who had continuous medical and prescription drug coverage from 3 months prior to the date of conception through 3 months post-delivery. We estimated the prevalence of opioid and benzodiazepine dispensing and co-dispensing before, during, and after pregnancy, and evaluated trends in dispensing patterns across the study period (2007–2015) using Cochrane-Armitage tests. Chi-square tests were used to examine differences in demographic and clinical characteristics by dispensing and co-dispensing patterns. Among women that received an opioid or benzodiazepine during pregnancy, logistic regression models were used to quantify the association between sample characteristics and dispensing patterns (co-dispensing vs single dispensing).

**Results:**

Of 168,025 pregnant women that met our inclusion criteria, 10.1% received at least one opioid and 2.0% received at least one benzodiazepine during pregnancy, while 0.5% were co-dispensed these drugs. During the study period (2007 vs 2015), prevalence of opioid dispensing during pregnancy decreased from 11.2 to 8.6% (*p* <  0.01); while benzodiazepine dispensing increased from 1.3 to 2.9% (*p* <  0.01), and the prevalence of co-dispensing, while low and stable, increased slightly from 0.39 to 0.44% (*p* <  0.01). Older age, a higher comorbidity burden, pain diagnosis, anxiety diagnosis, and alcohol, tobacco, and drug use disorders, were all associated with an increased odds of co-dispensing during pregnancy.

**Conclusions:**

This study provides evidence that while opioid dispensing during pregnancy has decreased in the past decade, benzodiazepine dispensing has increased. The prevalence of opioid-benzodiazepine co-dispensing was rare and remained fairly stable during our study period. Those co-dispensed both drugs had a higher prevalence of adverse birth outcomes. Further research to establish the potentially causal relationship between opioid and benzodiazepine co-dispensing and adverse birth outcomes should be undertaken.

**Supplementary Information:**

The online version contains supplementary material available at 10.1186/s12884-021-03787-5.

## Background

The use and co-use of opioids and benzodiazepines may pose significant health risks to pregnant women. Opioid use during pregnancy is associated with obstetric complications like spontaneous abortions and preeclampsia, as well as adverse birth outcomes such as neural tube defects, gastroschisis, and neonatal abstinence syndrome (NAS) [1–3]. While benzodiazepines may have health benefits that outweigh their risks [4], evidence on the safety of benzodiazepine use during pregnancy is mixed [5–9]. Some studies report that benzodiazepine use during pregnancy is associated with preterm birth, low birth weight, as well as congenital malformations [5–7], while other studies have found no impact of in-utero exposure to benzodiazepines on birthweight or neurodevelopment [8, 9]. The co-use of opioids and benzodiazepines in pregnancy is associated with an increased risk of maternal overdose and mortality, increased risk and severity of NAS, and longer newborn hospital stays [10–13].

The prevalence of opioid dispensing during pregnancy in the United States (U.S.) is well understood [14, 15]. Most recently, a study analyzing a nationally representative sample of commercially insured women reported a decreasing trend in opioid dispensing during pregnancy (14.9% in 2005 to 12.9% in 2011). However, the overall prevalence of dispensing was observed to be high at an average of 14.4% across the study period [14].

In contrast to opioids, the epidemiology of benzodiazepine dispensing during pregnancy has been less explored. The National Birth Defects Prevention Study, conducted across 10 U.S. states between 1997 and 2011, found that the prevalence of self-reported benzodiazepine use during pregnancy was rare (0.8%) [7]. Another study, in a commercially insured population, reported the prevalence of anxiolytic (benzodiazepine or Z-drug) dispensing during pregnancy to be 3.9% between 2006 and 2011 [16]. These prior studies have limited generalizability because of the clinically unique and/or geographically distinct study samples used, or are estimates of overall anxiolytic prescribing and not specifically of benzodiazepines [7, 16].

There is also limited literature on the co-dispensing of opioids and benzodiazepines during pregnancy. Investigating such dispensing patterns is especially important given that in 2016, the U.S. Food and Drug Administration (FDA) announced labelling changes, cautioning against the dangers of using opioids and benzodiazepines concomitantly [17]. Additionally, despite the risk for fatal overdose, recent evidence in the general population has found that over one-third of benzodiazepine prescriptions involved an overlapping opioid prescription [18].

In light of the research gaps and potential risks associated with use and co-use during pregnancy, we sought to quantify the prevalence and temporal trends of opioid and benzodiazepine dispensing and co-dispensing before, during, and after pregnancy, and by birth outcomes. Further, we examined differences in demographic and clinical characteristics of women in our sample by mutually exclusive opioid and benzodiazepine dispensing patterns.

## Methods

### Data source

In this retrospective study, we utilized a 10% random sample of primarily commercially insured (97%) enrollees in the IQVIA™ Adjudicated Health Plan Claims Data. While we only had access to 10% of the full data available through IQVIA, the sample was randomly generated by the vendor. Thus, to the extent possible, we believe the prevalence estimates are representative of the entire sample. IQVIA™ data is a longitudinal dataset and includes medical, pharmacy, and eligibility information from over 70 contributing health plans and self-insured employer groups throughout the U.S. for more than 140 million unique enrollees younger than 65 years of age.

### Study population

We identified women (12–55 years of age at date of delivery) with a completed pregnancy between 2007 and 2015. We used a previous algorithm (using ICD-9-CM diagnostic and procedure codes) for the identification of completed pregnancies, their categorization by birth outcomes (full-term birth, preterm birth, postterm birth, stillbirth), and the assignment of gestational age estimates [19]. We excluded women with spontaneous abortions or terminated pregnancies. In order to analyze drug dispensing patterns, beneficiaries were required to have continuous medical and prescription drug coverage from 3 months prior to the date of conception through 3 months post-delivery, allowing for a one-month gap in coverage. Only the first eligible delivery episode for each pregnant woman was considered for analysis.

### Definition of dispensing periods

We examined opioid and benzodiazepine dispensing and co-dispensing in each of the following periods: pre-conception (90-day period prior to the estimated date of conception); trimester 1 (estimated conception date through day 90 of pregnancy); trimester 2 (following 90 days); trimester 3 (beginning 181 days after estimated date of conception to date of delivery); and post-delivery (90-day period after the date of delivery).

### Definition of primary exposures

Our principal exposures of interest were opioid and benzodiazepine dispensing and co-dispensing. We only included opioids and benzodiazepines in the Centers for Disease Control and Prevention’s (CDC) guidance for analyzing opioid prescription data [20]. We excluded buprenorphine-naloxone combination products from the analysis. We used pharmacy claims to identify opioid and benzodiazepine dispensing dates within each time period of interest. The co-dispensing of opioids and benzodiazepines was defined based on prior literature as having at least a 1 day overlap in days’ supply of both drugs during any of the designated time periods [21].

### Demographic and clinical characteristics

The following demographic and clinical characteristics were determined on the date of delivery: age, region of residence, cesarean delivery. The following clinical characteristics were determined, using ICD-9-CM codes, between the start of the pre-conception period through the date of delivery: back pain, abdominal pain, arthritis pain, headache, cancer, obesity, anxiety, alcohol use disorder, tobacco use disorder, and drug use disorder. Finally, an obstetric comorbidity index, developed and validated previously, was used to determine the pre-pregnancy comorbidity burden over the same period [22].

### Statistical analysis

Descriptive analyses using chi-square tests were used to estimate demographic and clinical characteristics for the total sample and by each birth outcome. We estimated the prevalence of prescription opioid and benzodiazepine dispensing and co-dispensing before, during, and after pregnancy, and evaluated temporal trends during the study period as defined by delivery year using Cochrane-Armitage tests for trends.

Differences in beneficiary characteristics were also examined across five mutually exclusive groups depending on dispensing patterns during pregnancy: co-dispensed opioid-benzodiazepine, dispensed opioid only, dispensed benzodiazepine only, no opioid or benzodiazepine dispensing, and opioid-benzodiazepine dispensing but not co-dispensed. The latter group was defined as individuals who were dispensed both benzodiazepines and opioids at some point during pregnancy, but not concomitantly. Among women that received an opioid or benzodiazepine during pregnancy, a multivariable logistic regression was used to quantify the association between the sample characteristics and dispensing patterns. In this logistic regression analysis we compared those with co-dispensing vs single dispensing (opioid only, benzodiazepine only, opioid and benzodiazepine but not co-dispensed). The sample characteristics included in the model were age at delivery, region of residence, delivery year, comorbidity index score, obesity, cancer, any pain diagnosis (back pain, abdominal pain, arthritis pain, or headache), anxiety diagnosis, and alcohol, tobacco, or drug disorder diagnosis. All analyses were conducted using SAS version 9.4 and statistical significance was set at *α* = 0.05. The study received Institutional Review Board approval by the University of Maryland, Baltimore.

## Results

We identified 168,025 completed pregnancies over a nine-year period that met our eligibility criteria. Table 1 summarizes the demographic and clinical characteristics of the total sample and by individual birth outcomes. Birth outcomes in our sample were distributed as follows: full-term birth (79.4%), postterm birth (12.4%), preterm birth (7.2%), and stillbirth (1%). Relative to those with a full-term birth, women who experienced a preterm birth or a stillbirth were older at delivery, had a higher comorbidity burden, and displayed a higher prevalence of alcohol, tobacco, and drug use disorders.

**Table 1:** Demographic and clinical characteristics of commercially insured pregnant women (12–55 years of age) in the United States by birth outcome, 2007–2015

	Total population *N* = 168,025 (100%)		Full-term birth *N* = 133,459 (79.4%)		Preterm birth *N* = 12,166 (7.2%)		Postterm birth *N* = 20,802 (12.4%)		Stillbirth *N* = 1598 (1.0%)		*P*-value
	N	Col%	N	Col%	N	Col%	N	Col%	N	Col%	
**Age category, years**											< 0.01
12–17	1920	1.1	1452	1.1	165	1.4	280	1.3	23	1.4	
18–25	31,591	18.8	24,731	18.5	2276	18.7	4287	20.6	297	18.6	
26–34	97,905	58.3	77,734	58.3	6794	55.8	12,536	60.3	841	52.6	
≥ 35	36,609	21.8	29,542	22.1	2931	24.1	3699	17.8	437	27.4	
**Region**											< 0.01
East	35,220	21.0	27,042	20.2	2460	20.2	5404	25.9	314	19.6	
Midwest	52,909	31.5	41,321	31.0	3584	29.5	7527	36.2	477	29.9	
South	54,863	32.6	45,333	34.0	4293	35.3	4635	22.3	602	37.7	
West	25,033	14.9	19,763	14.8	1829	15.0	3236	15.6	205	12.8	
**Comorbidity score**											< 0.01
0	79,460	47.3	61,669	46.2	4078	33.5	13,080	62.9	633	39.6	
1	39,959	23.8	33,299	25.0	2075	17.1	4298	20.7	287	18.0	
≥ 2	48,606	28.9	38,491	28.8	6013	49.4	3424	16.4	678	42.4	
**Cesarean delivery**											< 0.01
Yes	40,749	24.3	33,412	25.0	8644	71.1	3615	17.4	200	12.5	
No	127,276	75.7	100,047	75.0	3522	28.9	17,187	82.6	1398	87.5	
**Obesity**											< 0.01
Yes	12,708	7.6	9987	7.5	1147	9.4	1418	6.8	156	9.8	
No	155,317	92.4	123,472	92.5	11,019	90.6	19,384	93.2	1442	90.2	
**Cancer**											0.49
Yes	1270	0.8	1000	0.8	106	0.9	152	0.7	12	0.7	
No	166,755	99.2	132,459	99.2	12,060	99.1	20,650	99.3	1586	99.3	
**Back pain**											< 0.01
Yes	30,441	18.1	24,121	18.1	2296	18.9	3785	18.2	239	15.0	
No	137,584	81.9	109,338	81.9	9870	81.1	17,017	81.8	1359	85.0	
**Abdominal pain**											< 0.01
Yes	37,766	22.5	29,851	22.4	3491	28.7	4045	19.4	379	23.7	
No	130,259	77.5	103,608	77.6	8675	71.3	16,757	80.6	1219	76.3	
**Arthritis pain**											< 0.01
Yes	35,090	20.9	27,645	20.7	2653	21.8	4478	21.5	314	19.7	
No	132,935	79.1	105,814	79.3	9513	78.2	16,324	78.5	1284	80.3	
**Headache**											< 0.01
Yes	6564	3.9	5171	3.9	575	4.7	748	3.6	70	4.4	
No	161,461	96.1	128,288	96.1	11,591	95.3	20,054	96.4	1528	95.6	
**Anxiety disorders**											< 0.01
Yes	10,059	6.0	7961	6.0	863	7.1	1121	5.4	114	7.1	
No	157,966	94.0	125,498	94.0	11,303	92.9	19,681	94.6	1484	92.9	
**Alcohol use disorder**											0.01
Yes	295	0.2	214	0.2	29	0.2	46	0.2	6	0.4	
No	167,730	99.8	133,245	99.8	12,137	99.8	20,756	99.8	1592	99.6	
**Tobacco use disorder**											< 0.01
Yes	7614	4.5	6047	4.5	720	5.9	769	3.7	78	4.9	
No	160,411	95.5	127,412	95.5	11,446	94.1	20,033	96.3	1520	95.1	
**Drug use disorder**											0.02
Yes	342	0.2	249	0.2	30	0.3	58	0.3	5	0.3	
No	167,683	99.8	133,210	99.8	12,136	99.7	20,744	99.7	1593	99.7	

**Fig. 1:**
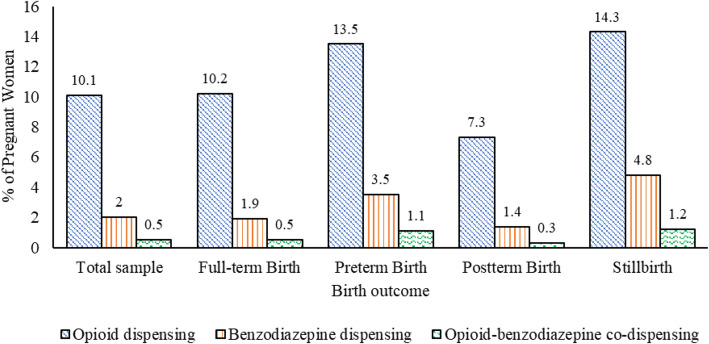
Opioid and benzodiazepine dispensing and co-dispensing during pregnancy by birth outcome among commercially insured women in the United States, 2007–2015. Statistically significant difference (*p* <  0.01) for opioid dispensing, benzodiazepine dispensing, and opioid-benzodiazepine co-dispensing by birth outcome

Figure 1 displays prevalence estimates of opioid and benzodiazepine dispensing and co-dispensing during pregnancy, in the total sample and by each birth outcome. Overall, 10.1% of the total sample was dispensed at least one opioid during pregnancy. Prevalence of opioid dispensing varied significantly (*p* <  0.01) by birth outcome: stillbirth (14.3%), preterm birth (13.5%), full-term birth (10.2%), and postterm birth (7.3%). Two percent of the total sample was dispensed at least one benzodiazepine during pregnancy. The prevalence of benzodiazepine dispensing varied significantly (*p* <  0.01) by birth outcome: stillbirth (4.8%), preterm birth (3.5%), full-term birth (1.9%), and postterm birth (1.4%). Finally, 0.5% of the total sample was co-dispensed opioids and benzodiazepines during pregnancy. Prevalence of co-dispensing also varied significantly (*p* <  0.01) by birth outcome: stillbirth (1.2%), preterm birth (1.1%), full-term birth (0.5%), and postterm birth (0.3%).

**Fig. 2:**
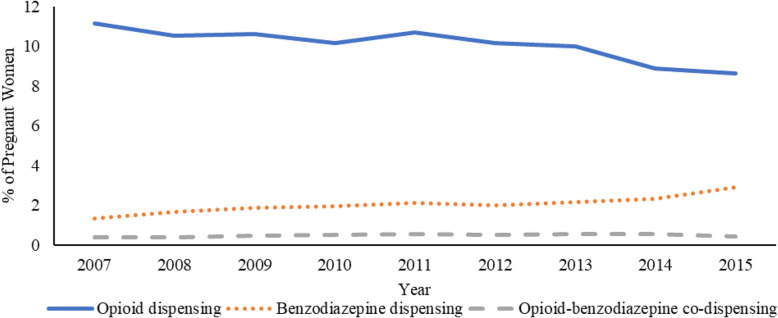
Temporal trends in opioid and benzodiazepine dispensing and co-dispensing during pregnancy among commercially insured women in the United States, 2007–2015. Statistically significant test of linear trend for opioid dispensing (*p* <  0.01), benzodiazepine dispensing (*p* <  0.01), and opioid-benzodiazepine co-dispensing (*p* = 0.01)

Figure 2 reflects temporal trends in opioid and benzodiazepine dispensing as well as co-dispensing during pregnancy across each year of the study for the total sample. While there was a statistically significant decrease in the prevalence of opioid dispensing during pregnancy between 2007 and 2015 (11.2 to 8.6%, *p* <  0.01), benzodiazepine dispensing increased over the same period (1.3 to 2.9%, *p* <  0.01). In addition, there was a statistically significant increase in opioid-benzodiazepine co-dispensing during pregnancy over the same period (0.39% vs 0.44%, *p* = 0.01). Additional file 1 reflects the statistically significant increasing trend in the prevalence of any pain diagnosis (back pain, abdominal pain, arthritis pain or headache), anxiety diagnosis, and cesarean delivery across the study period in the total sample. Additional file 2 and Additional file 3 present temporal trends for the five most commonly dispensed opioids and benzodiazepines, respectively, during pregnancy across the study period. Additional file 4 and Additional file 5 describe the prevalence of the five most commonly dispensed opioids and benzodiazepines, respectively, before, during, and after pregnancy.

**Fig. 3:**
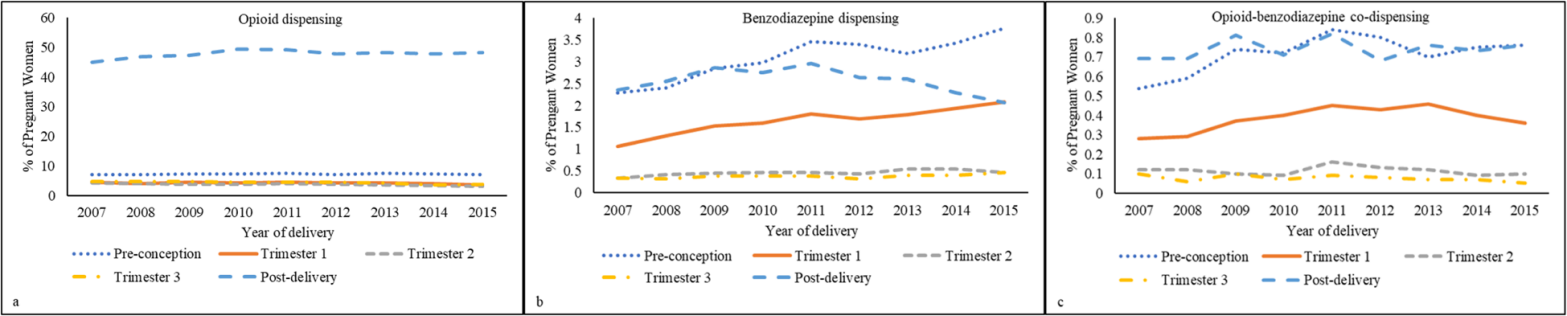
Temporal trends in opioid and benzodiazepine dispensing and co-dispensing by pregnancy time periods among commercially insured women in the United States, 2007–2015. Statistically significant test of linear trend for opioid dispensing during all time periods (p <  0.01); benzodiazepine dispensing during pre-conception (*p*< 0.01), 1st trimester (*p*< 0.01), 2nd trimester (*p*< 0.01), and post-delivery (*p*< 0.01) periods; and opioid-benzodiazepine co-dispensing during pre-conception (*p* = 0.01) and 1st trimester (*p* = 0.01) periods

Figures 3a-c display temporal trends in opioid and benzodiazepine dispensing and co-dispensing by delivery year and pregnancy period for the total sample. A significantly decreasing trend (2007 vs 2015) in opioid dispensing during the 1st trimester (4.6% vs 3.4%, *p* <  0.01), 2nd trimester (4.3% vs 3%, *p* <  0.01), and 3rd trimester (4.8% vs 3.7%, *p* <  0.01) was observed across the study period. Conversely, prevalence of opioid dispensing during the post-delivery period increased over the same period (45.0% vs 48.3%, *p* <  0.01) (Fig. 3a).

The prevalence of benzodiazepine dispensing increased (2007 vs 2015) in the pre-conception (2.3% vs 3.8%, *p* <  0.01), 1st trimester (1.1% vs 2.1%, *p* <  0.01), and 2nd trimester (0.3% vs 0.5%, *p* <  0.01) periods. Conversely, dispensing in the post-delivery period decreased over a similar time period (2.4% vs 2.1%, *p* = 0.02) (Fig. 3b). The prevalence of opioid-benzodiazepine co-dispensing increased (2007 vs 2015) in the pre-conception (0.5% vs 0.8%, *p* = 0.01) and 1st trimester (0.3% vs 0.4%, *p* = 0.01) periods (Fig. 3c). Additional file 6 describes trends (2007 vs 2015) in opioid dispensing and opioid-benzodiazepine co-dispensing patterns in the post-delivery period by mode of delivery (cesarean vs vaginal). Table 2 describes demographic and clinical characteristics of the sample by mutually exclusive opioid and benzodiazepine dispensing and co-dispensing patterns. Relative to those dispensed an opioid only, women co-dispensed opioids and benzodiazepines during pregnancy were older at birth, primarily resided in the south region of the U.S., had a higher comorbidity burden and displayed a higher prevalence of alcohol, tobacco, or drug use disorders.

**Table 2:** Demographic, clinical, and birth outcome characteristics of commercially insured pregnant women in the United States (12–55 years of age) by mutually exclusive opioid and benzodiazepine dispensing categories, 2007–2015

	Co-dispensed opioid-benzodiazepine *N* = 808 (0.5%)		Dispensed, not co-dispensed, opioid- benzodiazepine *N* = 476 (0.3%)		Dispensed opioid only *N* = 15,699 (9.3%)		Dispensed benzodiazepine only *N* = 2035 (1.2%)		Not dispensed opioid or benzodiazepine *N* = 149,007 (88.7%)		*P*-value
	N	%	N	%	N	%	N	%	N	%	
**Age category, years**											< 0.01
12–25	98	12.1	68	14.3	3723	23.7	251	12.3	29,371	19.7	
26–34	459	56.8	275	57.8	8805	56.1	1071	52.6	87,295	58.6	
≥ 35	251	31.1	133	27.9	3171	20.2	713	35.1	32,341	21.7	
**Region**											< 0.01
East	153	18.9	82	17.2	2322	14.8	480	23.6	32,183	21.6	
Midwest	176	21.8	134	28.2	4799	30.6	589	28.9	47,211	31.7	
South	349	43.2	215	45.2	6111	38.9	771	37.9	47,417	31.8	
West	130	16.1	45	9.4	2467	15.7	195	9.6	22,196	14.9	
**Comorbidity index**											< 0.01
0	236	29.2	142	29.8	6438	41.0	673	33.0	71,971	48.3	
1	197	24.4	111	23.3	3888	24.8	500	24.6	35,263	23.7	
≥ 2	375	46.4	223	46.9	5373	34.2	862	42.4	41,773	28.0	
**Cesarean delivery**											< 0.01
Yes	296	36.6	164	34.5	4484	28.6	638	31.4	35,167	23.6	
No	512	63.4	312	65.5	11,215	71.4	1397	68.6	113,840	76.4	
**Birth type**											< 0.01
Full-term birth	601	74.4	366	76.9	12,625	80.4	1561	76.7	118,306	79.4	
Preterm birth	132	16.3	56	11.8	1450	9.2	232	11.4	10,296	6.9	
Post term birth	56	6.9	41	8.6	1427	9.1	197	9.7	19,081	12.8	
Stillbirth	19	2.4	13	2.7	197	1.3	45	2.2	1324	0.9	
**Obesity**											< 0.01
Yes	100	12.4	67	14.1	1691	10.8	213	10.5	10,637	7.1	
No	708	87.6	409	85.9	14,008	89.2	1822	89.5	138,370	92.9	
**Cancer**											< 0.01
Yes	13	1.6	9	1.9	172	1.1	30	1.5	1046	0.7	
No	795	98.4	467	98.1	15,527	98.9	2005	98.5	147,961	99.3	
**Back pain**											< 0.01
Yes	384	47.5	197	41.4	5066	32.3	458	22.5	24,336	16.3	
No	424	52.5	279	58.6	10,633	67.7	1577	77.5	124,671	83.7	
**Abdominal pain**											< 0.01
Yes	340	42.1	220	46.2	6790	43.3	590	29.0	29,826	20.0	
No	468	57.9	256	53.8	8909	56.7	1445	71.0	119,181	80.0	
**Arthritis pain**											< 0.01
Yes	372	46.0	198	41.6	5102	32.5	604	29.7	28,814	19.3	
No	436	54.0	278	58.4	10,597	67.5	1431	70.3	120,193	80.7	
**Headache**											< 0.01
Yes	149	18.4	70	14.7	1678	10.7	124	6.1	4543	3.1	
No	659	81.6	406	85.3	14,021	89.3	1911	93.9	144,464	96.9	
**Anxiety disorders**											< 0.01
Yes	325	40.2	198	58.4	1338	8.5	855	42.0	7343	4.9	
No	483	59.8	278	41.6	14,361	91.5	1180	58.0	141,664	95.1	
**Alcohol, tobacco, or drug use disorder**											< 0.01
Yes	159	19.7	69	14.5	1659	10.6	202	9.9	6058	4.1	
No	649	80.3	407	85.5	14,040	89.4	1833	90.1	142,949	95.9	

**Fig. 4:**
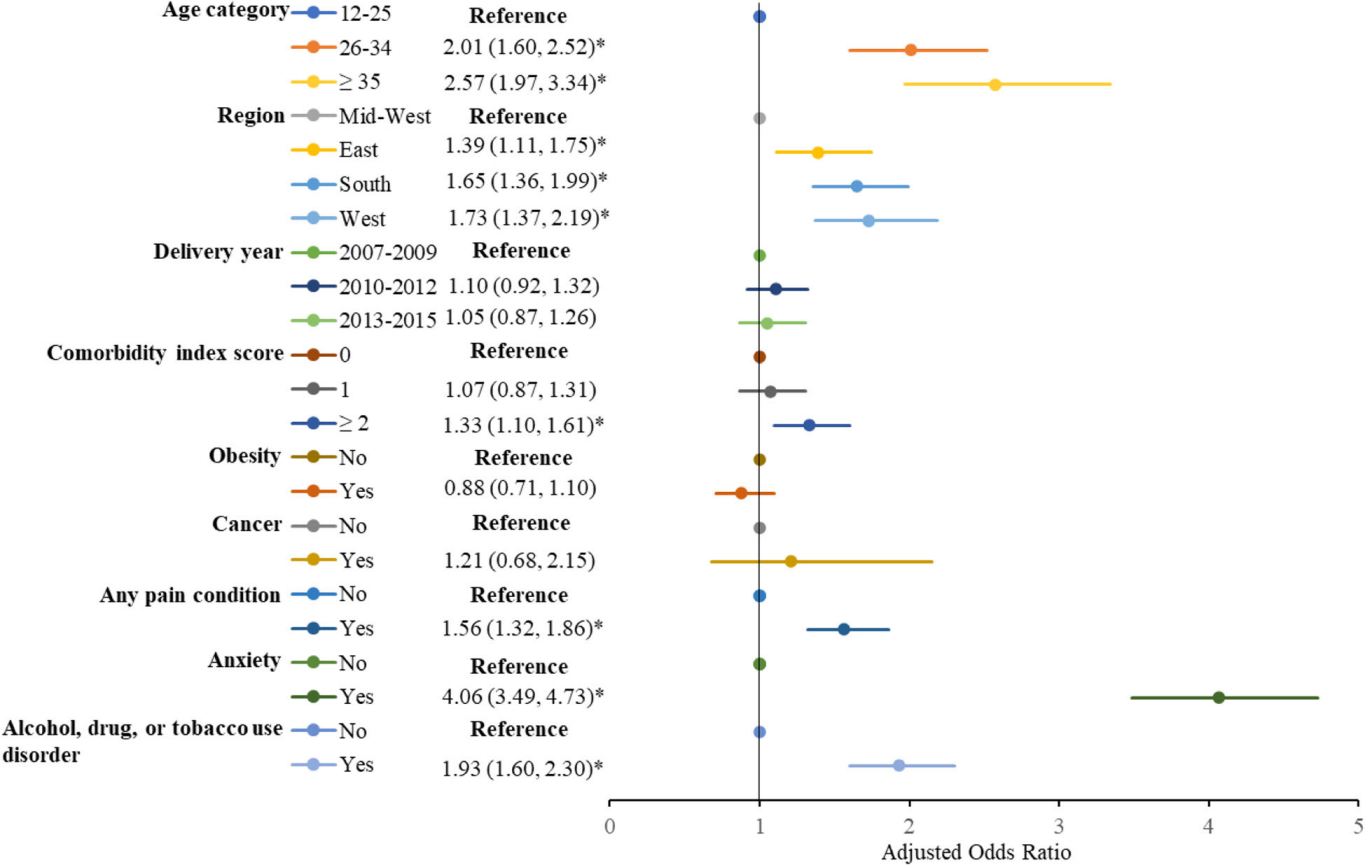
Multivariable logistic regression analysis of demographic and clinical factors associated with opioid-benzodiazepine co-dispensing during pregnancy among commercially insured women in the United States, 2007–2015. *Indicates statistical significance at *p* <  0.01; Adjusted odds ratio > 1 indicates greater odds of co-dispensing versus single dispensing; Adjusted odds ratio < 1 indicates lower odds of co-dispensing versus single dispensing

Figure 4 presents the adjusted odds ratios (AOR) and 95% confidence intervals (CI) from the logistic regression model predicting opioid-benzodiazepine co-dispensing vs single dispensing. Determinants of co-dispensing during pregnancy include: older age, specifically women aged ≥35 years compared to those 18–25 years [AOR(95% CI): 2.57 (1.97, 3.34)]; residence in the south region of the U.S. compared to midwest region [AOR 1.65 (1.36, 1.99)]; a higher comorbidity index score (≥ 2 vs 0) [AOR 1.33 (1.10, 1.61)]; having a pain diagnosis [AOR 1.56 (1.32, 1.86)]; having an anxiety diagnosis [AOR 4.06 (3.49, 4.73)]; and having an alcohol, tobacco, or drug use disorder [AOR 1.93 (1.60, 2.30)].

## Discussion

Over our 9-year study period, 1 in 10 and 1 in 50 pregnant women were dispensed an opioid and benzodiazepine, respectively, during pregnancy. We observed a decreasing trend in opioid dispensing and an increasing trend in benzodiazepine dispensing during pregnancy. The prevalence of opioid-benzodiazepine co-dispensing during pregnancy was rare and remained stable over time. Sample characteristics such as older age, geographic residence, a higher comorbidity index score, presence of a pain diagnosis, presence of an anxiety diagnosis, and presence of alcohol, tobacco, and drug use disorders were significantly associated with opioid-benzodiazepine co-dispensing during pregnancy. Women who received both drugs had a higher prevalence of adverse birth outcomes compared to those dispensed opioids or benzodiazepines alone.

Our estimated prevalence of opioid dispensing during pregnancy of 10.1%, is lower than that previously reported in a commercially insured population (14.4%) in an earlier time period (2005–2011) [14]. Conversely, the prevalence of opioid dispensing in our study was higher than that observed in a European population in an earlier time period. A prior study using the linked Norwegian Prescription-Birth Registry Database reported the prevalence of opioid dispensing to be 3% during pregnancy between 2004 and 2009 [23]. Our study findings thus reflect the decreasing trend in opioid use in the general U.S. population since 2012 [24]. Of note, despite the decrease in opioid dispensing, we found an increasing trend in pain diagnosis during the study period. The decreasing trend in opioid dispensing may thus be explained, in part, by the concerted federal, state, and local public health and managed care initiatives which have targeted the reduction of opioid prescribing rates in the general population in recent years [25–27]. Further, opioid dispensing during the post-delivery period rose significantly during our study period (2007–2015). This may be attributable to the overall increase (19.8 to 23.9%) in cesarean deliveries in our sample as well as in the U.S during our study period [28].

Compared to our estimates, the National Birth Defects Prevention (NBDP) study reported a lower prevalence of self-reported benzodiazepine use during pregnancy (0.8%) between 1997 and 2011 [7]. Notably, the NBDP study excluded states such as Alabama, Tennessee, and West Virginia, reported to have comparatively higher benzodiazepine prescribing rates than the general U.S. population [29]. Our estimate (2%) for benzodiazepine dispensing during pregnancy was similar to that (1.9%) reported in a prior meta-analysis which included 32 studies across 28 countries [30]. In sharp contrast to trends in opioid use, the prevalence of benzodiazepine dispensing during pregnancy more than doubled during our study period. Anxiety, the most frequent condition for which benzodiazepines are prescribed, is common and its prevalence is increasing among pregnant women [31]. Not surprisingly then, analogous to the trend in benzodiazepine dispensing, the prevalence of anxiety in our sample doubled (4.0 to 8.3%) during the study period. Benzodiazepine dispensing during pre-conception and all three trimesters also rose significantly during our study period, again, likely reflective of the overall increase in prevalence of anxiety diagnoses.

We found that the prevalence of opioid-benzodiazepine co-dispensing during pregnancy was rare (0.5%). One study, based on self-reported substance use among pregnant women admitted to opioid use disorder (OUD) treatment facilities, found that concomitant benzodiazepine use ranged from 3 to 13% depending on region of residence in the U.S. [32]. However, these individuals represent a subgroup with increased use patterns not representative of the general pregnant population. Another study estimated that the proportion of commercially insured individuals prescribed an opioid who were also prescribed a benzodiazepine in the general population (i.e. including men and non-pregnant women) was 17% in 2013 [21].

Not unexpectedly, while co-dispensing during pregnancy in our study was rare, variation was observed by birth outcomes. Pregnancies ending in preterm birth and stillbirth had 2- and 3-times higher prevalence of co-dispensing, respectively, compared to full-term birth. This finding should be considered with caution, as we did not study the causal association between co-dispensing and birth outcomes. Previous reports about the potential detrimental effects of these drugs on maternal and fetal health have been noted however [1–3, 5–7, 9]. Further, these findings are of concern given recent evidence that co-exposure to benzodiazepines and opioids increases the risk and severity of NAS [10, 11].

We found stable rates of co-dispensing during our study period. Though this cannot be attributed to the CDC guidelines and FDA drug labelling changes cautioning against co-use which were introduced later in 2016 [17, 33], it may be ascribed to similar guidelines developed earlier by the American Geriatrics Society and the American Society of Interventional Pain Physicians [34, 35]. While these guidelines are not specific to pregnant women, they helped create awareness of the risks of combined use, among both prescribers as well as patients.

In our sample and relative to those dispensed an opioid only, women co-dispensed opioids and benzodiazepines during pregnancy were older at birth, had a higher comorbidity burden, and displayed a higher prevalence of alcohol, tobacco, and drug use disorders. These findings are in line with a prior study analyzing a publicly insured U.S. sample of pregnant women co-prescribed opioids and psychotropics [10]. Similarly, our results from the logistic regression found that older age, geographic residence, a higher comorbidity index score, and presence of alcohol, tobacco, and drug use disorders were significantly associated with opioid-benzodiazepine co-dispensing (vs single dispensing of either product) during pregnancy. Pain and anxiety diagnosis were also found to be associated with co-dispensing during pregnancy.

Geographically, we found a higher prevalence of opioid and benzodiazepine dispensing and co-dispensing in the south compared to other U.S. regions. Prior studies have reported opioid prescribing rates are highest among commercially insured women of reproductive age residing in the south [36]. While similar estimates for benzodiazepine prescribing among pregnant women are not known, our results are consonant with prior findings of higher benzodiazepine prescribing rates in the south among commercially insured adults as compared to other U.S. regions [29]. Further, our findings reaffirm conclusions from a prior study which also found higher co-use of opioids and benzodiazepines in the south among pregnant women entering OUD treatment facilities [32].

The increasing use of benzodiazepines is compelling to consider as a parallel problem to the opioid crisis [37]. Despite their associated risks, benzodiazepine use in vulnerable populations such as pregnant women has received little attention among clinicians, policymakers, and educators. Given the persistent co-use of opioids and benzodiazepines in the general population, as evidenced by recent research [18], our findings underscore the need to expand the focus of educational and intervention programs aimed at improving safe medication use among pregnant women beyond opioids. While opioids have become the focus of such programs, their aims should also extend to promoting prudent use of benzodiazepines and mitigating co-use of benzodiazepines and opioids.

Given the potential harmful effects of benzodiazepines on maternal and fetal health, our findings also highlight the need for non-pharmacological interventions to help relieve anxiety during pregnancy [38]. Removing barriers to access and improving insurance reimbursement for these interventions may help mitigate potentially inappropriate benzodiazepine prescribing in this population.

Given the lack of research on the safety of benzodiazepine use during pregnancy, our findings indicate several directions for future research. First, there is a need for additional studies to confirm the generalizability of our results in publicly insured and uninsured pregnant women. Second, future research should extend beyond dispensing patterns, to understand specific benzodiazepine drug doses or combinations that best balance the benefits of managing anxiety with the potential risk for adverse maternal and fetal outcomes. Third, in addition to prescribing guidelines and education, there is a need to evaluate additional strategies to manage or reduce the dispensing and co-dispensing of these drugs. Such strategies may include addressing benzodiazepine tapering or dose modification during pregnancy [39, 40], improving treatment of comorbid behavioral and mental health issues, and testing the comparative effectiveness of non-pharmacologic interventions. A comprehensive analysis of additional strategies will better inform policy and program development aimed at improving safe medication use during pregnancy.

Our study had several strengths. Our national sample allowed us to investigate opioid and benzodiazepine dispensing patterns in a cohort of 168,025 completed pregnancies sampled from over 70 commercial health plans across the U.S., with fully adjudicated medical and pharmacy claims. Whereas prior studies were mainly limited to live births, we included all completed pregnancy birth outcomes. Our study period was expansive and spanned 9 years, allowing for the examination of time trends in patterns before, during, and after pregnancy. Our focus on a commercially insured population allowed for a comprehensive analysis of prescribing practices in this population.

The present study had certain limitations. Importantly, our findings for the descriptive and trend analysis should be considered in light of the large sample size, which may yield statistically significant *p*-values for results that may not be of clinical significance. Further, our findings may not be generalizable to a large proportion of pregnant women who are uninsured or publicly insured. In addition, our findings are based on pharmacy dispensing claims that may not necessarily reflect use or prescribing of medications. Hence, we may have underestimated patterns of use for individuals who did not fill their prescriptions. Conversely, we may have overestimated patterns of use for individuals who were non-adherent to their medication regimen. We were also unable to capture individuals who may be using these drugs without a prescription. Finally, we imputed the date of conception based on claims for labor and delivery, which provide information on birth outcomes. Although this approach has been used previously, some degree of misclassification of pregnancy period is possible [19].

## Conclusion

The present study evaluated real world dispensing patterns of benzodiazepines, both singularly and in combination with opioids, in a national sample of commercially insured pregnant women. Our findings indicate that as clinicians become increasingly cautious of prescribing opioids during pregnancy, benzodiazepine prescribing continues to increase. Our findings highlight the need for education and development of clinical guidelines for the safe use of benzodiazepines alone, and in combination with opioids, during pregnancy. Considering evidence suggesting the potentially teratogenic impact of these agents in combination, and especially given the paucity of safety evidence supporting their use, the persistent use of both agents, alone or in combination, is a health concern that warrants further attention.

## Supplementary Information

**Additional file 1.** Temporal trends in pain diagnosis, cesarean delivery and anxiety diagnosis during pregnancy, 2007–2015. This figure displays temporal trends in pain diagnosis, cesarean delivery, and anxiety diagnosis among commercially insured pregnant women in the United States.

**Additional file 2.** Temporal trends for the five most commonly dispensed opioids during pregnancy, 2007–2015. This figure displays temporal trends in utilization of the five most commonly dispensed opioids during pregnancy among commercially insured pregnant women in the United States.

**Additional file 3.** Temporal trends for the five most commonly dispensed benzodiazepines during pregnancy, 2007–2015. This figure displays temporal trends in utilization of the five most commonly dispensed benzodiazepines during pregnancy among commercially insured pregnant women in the United States.

**Additional file 4.** Prevalence of the overall and the five most dispensed opioids before, during and after pregnancy, 2007–2015. This table displays prevalence in utilization of the five most commonly dispensed opioids before, during and after pregnancy among commercially insured pregnant women in the United States.

**Additional file 5.** Prevalence of the overall and the five most dispensed benzodiazepines before, during and after pregnancy, 2007–2015. This table displays prevalence in utilization of the five most commonly dispensed benzodiazepines before, during and after pregnancy among commercially insured pregnant women in the United States.

**Additional file 6.** Temporal trends in opioid dispensing and opioid-benzodiazepine co-dispensing in the post-delivery period by mode of delivery (cesarean delivery vs vaginal delivery) in the United States, 2007–2015. This figure displays the trends for opioid dispensing and opioid-benzodiazepine co-dispensing in the post-delivery period by mode of delivery among commercially insured women in the United States.

## Data Availability

The data utilized in this analysis was obtained under license from IQVIA and is not publicly available.

## Abbreviations

NAS: Neonatal abstinence syndrome
FDA: Food and Drug Administration
CDC: Centers for Disease Control and Prevention
NBDP: National Birth Defects Prevention
OUD: Opioid Use Disorder
