# Supplementary material for: Opioid and benzodiazepine dispensing and co-dispensing patterns among commercially insured pregnant women in the United States, 2007–2015
Source: BMC Pregnancy Childbirth. 2021 May 3;21:350. doi: 10.1186/s12884-021-03787-5 (PMC8091773; doi:10.1186/s12884-021-03787-5)
Supplement: Supplementary file 1 — Additional file 1. Temporal trends in pain diagnosis, cesarean delivery and anxiety diagnosis during pregnancy, 2007–2015. This figure displays temporal trends in pain diagnosis, cesarean delivery, and anxiety diagnosis among commercially insured pregnant women in the United States. [file 12884_2021_3787_MOESM1_ESM.docx]

**File name:** Additional File 1

**Title:** Temporal trends in pain diagnosis, cesarean delivery and anxiety diagnosis during pregnancy, 2007-2015

**Description:** This figure displays temporal trends in pain diagnosis, ceseraen delivery, and anxiety diagnosis during pregnancy among commercially insured pregnant women in the United States


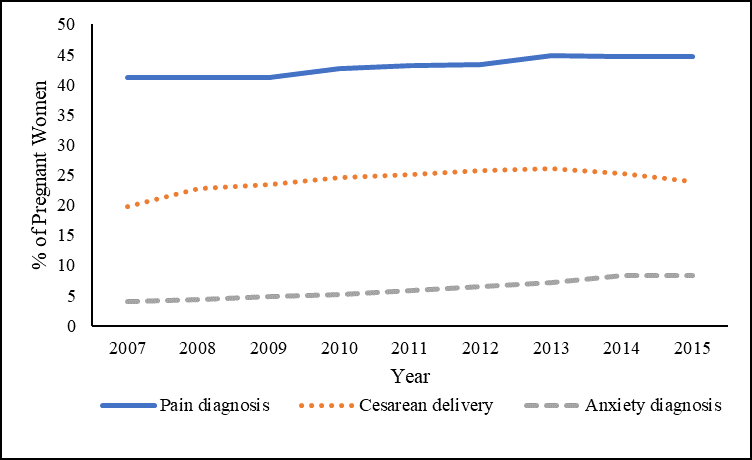


Pain diagnosis (2007 vs 2015): 41.1% to 44.6%, p<0.01

Cesarean delivery (2007 vs 2015): 19.8% to 23.9%, p<0.01

Anxiety diagnosis (2007 vs 2015): 4% to 8.3%, p<0.01
