# Supplementary material for: Opioid and benzodiazepine dispensing and co-dispensing patterns among commercially insured pregnant women in the United States, 2007–2015
Source: BMC Pregnancy Childbirth. 2021 May 3;21:350. doi: 10.1186/s12884-021-03787-5 (PMC8091773; doi:10.1186/s12884-021-03787-5)
Supplement: Supplementary file 2 — Additional file 2. Temporal trends for the five most commonly dispensed opioids during pregnancy, 2007–2015. This figure displays temporal trends in utilization of the five most commonly dispensed opioids during pregnancy among commercially insured pregnant women in the United States. [file 12884_2021_3787_MOESM2_ESM.docx]

**File name:** Additional File 2

**Title:** Temporal trends for the five most commonly dispensed opioids during pregnancy, 2007-2015

**Description:** This figure displays temporal trends in utilization of the five most commonly dispensed opioids during pregnancy among commercially insured pregnant women in the United States.


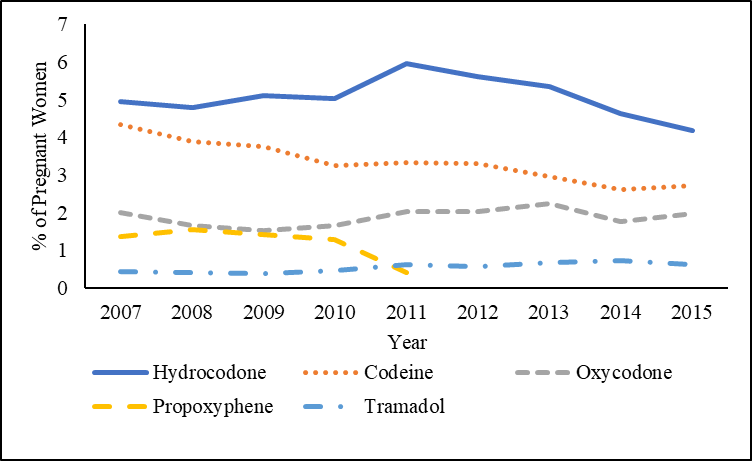


*Propoxyphene was discontinued in 2011

Hydrocodone (2007 vs 2015): 5% to 4.2%

Codeine (2007 vs 2015): 4.4% vs 2.7%

Oxycodone (2007 vs 2015): 2% vs 2%

Propoxyphene (2007 vs 2011): 1.4% vs 0.4%

Tramadol (2007 vs 2015): 0.4% vs 0.6%
