# Supplementary material for: Opioid and benzodiazepine dispensing and co-dispensing patterns among commercially insured pregnant women in the United States, 2007–2015
Source: BMC Pregnancy Childbirth. 2021 May 3;21:350. doi: 10.1186/s12884-021-03787-5 (PMC8091773; doi:10.1186/s12884-021-03787-5)
Supplement: Supplementary file 4 — Additional file 4. Prevalence of the overall and the five most dispensed opioids before, during and after pregnancy, 2007–2015. This table displays prevalence in utilization of the five most commonly dispensed opioids before, during and after pregnancy among commercially insured pregnant women in the United States. [file 12884_2021_3787_MOESM4_ESM.docx]

**File name:** Additional File 4

**Title:** Prevalence of the overall and the five most commonly dispensed opioids before, during and after pregnancy, 2007-2015

**Description:** This table displays prevalence in utilization of the five most commonly dispensed opioids before, during and after pregnancy in commercially insured pregnant women in the United States.

|  | **Exposure Window** | | | | | |
| --- | --- | --- | --- | --- | --- | --- |
|  | **Pre-conception** | **First trimester** | **Second trimester** | **Third Trimester** | **Post-delivery** | **Anytime during pregnancy** |
|  | **N (%)** | **N (%)** | **N (%)** | **N (%)** | **N (%)** | **N (%)** |
| Any opioid | 12,199 (7.3) | 7,077 (4.2) | 6,337 (3.8) | 7,360 (4.4) | 76,941 (45.8) | 16,983 (10.1) |
| Specific opioid |  |  |  |  |  |  |
| Hydrocodone | 7,518 (4.5) | 3,870 (2.3) | 2,910 (1.7) | 3,372 (2.0) | 31,328 (18.6) | 8,608 (5.1) |
| Codeine | 1,459 (0.9) | 1,592 (1.0) | 2,251 (1.3) | 2,377 (1.4) | 7,941 (4.7) | 5,622 (3.4) |
| Oxycodone | 2,451 (1.5) | 1,145 (0.7) | 1,013 (0.6) | 1,423 (0.9) | 37,996 (22.6) | 3,156 (1.9) |
| Propoxyphene | 845 (0.5) | 117 (0.1) | 119 (0.1) | 445 (0.3) | 3,705 (2.2) | 1,129 (0.7) |
| Tramadol | 1,375 (0.8) | 717 (0.4) | 222 (0.1) | 194 (0.1) | 1,537 (0.9) | 920 (0.6) |
