# Supplementary material for: Opioid and benzodiazepine dispensing and co-dispensing patterns among commercially insured pregnant women in the United States, 2007–2015
Source: BMC Pregnancy Childbirth. 2021 May 3;21:350. doi: 10.1186/s12884-021-03787-5 (PMC8091773; doi:10.1186/s12884-021-03787-5)
Supplement: Supplementary file 6 — Additional file 6. Temporal trends in opioid dispensing and opioid-benzodiazepine co-dispensing in the post-delivery period by mode of delivery (cesarean delivery vs vaginal delivery) in the United States, 2007–2015. This figure displays the trends for opioid dispensing and opioid-benzodiazepine co-dispensing in the post-delivery period by mode of delivery among commercially insured women in the United States. [file 12884_2021_3787_MOESM6_ESM.docx]

**File name:** Additional File 6

**Title:** Temporal trends in opioid dispensing and opioid-benzodiazepine co-dispensing in the post-delivery period by mode of delivery (cesarean delivery vs vaginal delivery) in the United States, 2007-2015.

**Description:** This table displays the trends for opioid dispensing and opioid-benzodiazepine co-dispensing in the post-delivery period by mode of delivery among commercially insured women in the United States.


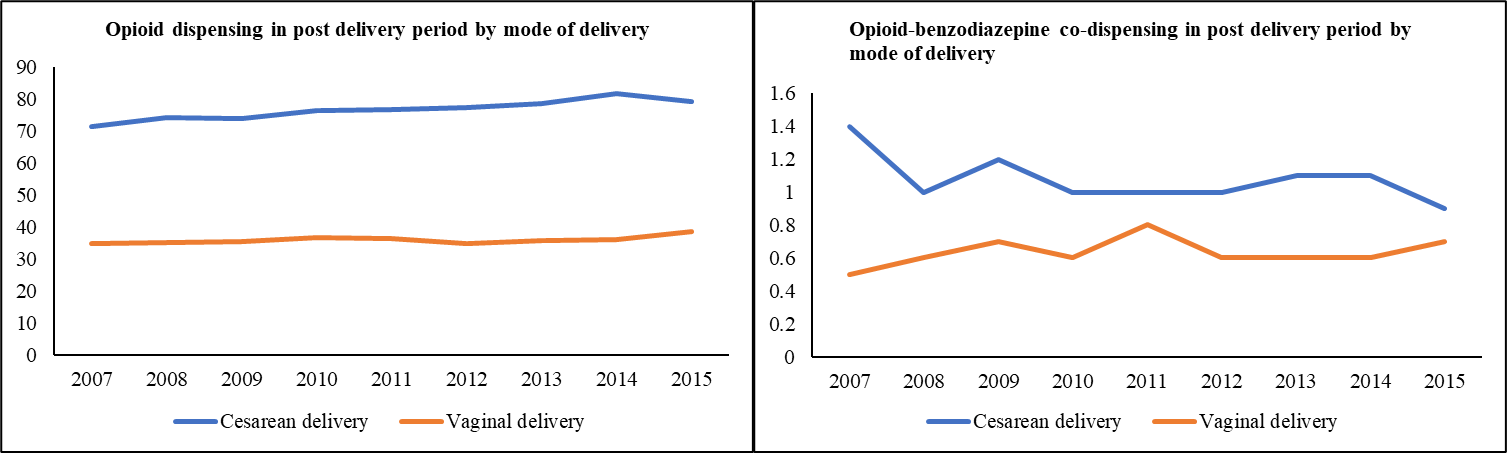


Trends (2007 vs 2015) in opioid dispensing during the post-delivery period among pregnant women with a cesarean delivery: 71.4% vs 79.3%

Trends (2007 vs 2015) in opioid dispensing during the post-delivery period for pregnant women with a vaginal delivery: 34.9% vs 38.6%

Trends (2007-2015) in opioid-benzodiazepine co-dispensing during in the post-delivery period among pregnant women with a cesarean delivery: 1.4% v 0.9%

Trends (2007-2015) in opioid-benzodiazepine co-dispensing during in the post-delivery period among pregnant women with a vaginal delivery: 0.5% vs 0.7%
